# Multi-Scale Assessment and Spatio-Temporal Interaction Characteristics of Ecosystem Health in the Middle Reaches of the Yellow River of China

**DOI:** 10.3390/ijerph192316144

**Published:** 2022-12-02

**Authors:** Wei Shen, Yang Li

**Affiliations:** 1 College of Land and Tourism, Luoyang Normal University, Luoyang 471022, China; 2 College of Geography and Environmental Science, Henan University, Kaifeng 475004, China; 3 Key Laboratory of Geospatial Technology for the Middle and Lower Yellow River Regions, Henan University, Kaifeng 475004, China

**Keywords:** improved VORS model, spatial weight coefficient, modified coefficient of spatial proximity effect, optimal analytical scale, ESTDA model

## Abstract

Exploring the assessment methods and multi-scale spatiotemporal interaction characteristics of ecosystem health is of great significance for current ecosystem health theory and application research. Based on the regional differentiation theory and ecosystem service flow theory, the spatial weight coefficient and the modified coefficient of spatial proximity effect were introduced to improve the regional ecosystem health assessment model. Then, the improved VORS model was used to evaluate the ecosystem health level in the Middle Reaches of the Yellow River (MRYR) in China at multiple scales, and the ESTDA method was used to reveal the multi-scale spatiotemporal interaction characteristics of ecosystem health. The results show that: (1) From 1990 to 2018, the ecosystem health level at grid and county scale in the MRYR showed a trend of first decline and then increase, and experienced a slow decline and a steady rise from 1990 to 2005 and 2005 to 2018, respectively. The ecosystem health level at the grid and county scale presented a spatially hierarchical structure with alternating low-value and high-value zones. (2) Compared with the county scale, the grid scale can describe the spatial distribution characteristics of ecosystem health more refined, indicating the existence of spatial scale effects in ecosystem health assessment. (3) The rapid urbanization areas, the ecologically fragile areas in the central and western regions and the transitional zone between mountain and basin have more dynamic spatial structure, and stronger spatio-temporal interaction process. (4) In terms of LISA spatio-temporal transition, the regional system as a whole had strong path-dependent and lock-in characteristics, and the local spatial correlation structure of ecosystem health gradually tended to be stable during the study period. (5) In terms of spatio-temporal interaction network, there were strong spatio-temporal competition in the process of time evolution in six typical regions, such as the surrounding cities of provincial capitals, the fringe areas of cities, the transitional zone between mountain and basin, the transitional zone of ecologically fragile regions, the mountainous areas of western Henan Province, and the areas along rivers.

## 1. Introduction

With the intensification of human activities, the problem of ecosystem health has become the main environmental problem faced by human beings [1,2,3]. The deterioration of ecosystem health will directly affect the service function of ecosystem and human access to and use of natural resources, and then pose a grievous menace to human health and the sustainable development of economic society [4,5,6]. Under the dual influence of climate and human activities, the ecologically fragile areas in Western China have produced soil erosion [7], water and soil environmental pollution [8,9,10], over exploitation of water resources [6,11,12], land desertification [13,14], vegetation degradation [15,16,17] and a series of eco-environmental problems, and seriously endanger the healthy development of regional ecosystem. In the context of promoting the eco-environmental protection and high-quality development strategy in the Yellow River Basin, this study scientifically assessed the ecosystem health level in the Middle Reaches of the Yellow River, and explored the multi-scale spatial pattern and spatio-temporal interaction characteristics of ecosystem health, which has important theoretical and practical significance for environmental management and regional sustainable development.

Ecosystem health refers to the sustainability and self-maintenance ability of ecosystem chimeras and the stability of ecosystem services provided at a certain spatial and temporal scale [18,19,20]. The exploration of ecosystem health assessment methods began in the late 1980s. According to the proposed chronological order, the comprehensive indicator method mainly includes the vigor-organization-resilience (VOR) model [18,20], subsystem model, pressure-state-response (PSR) model [21], and the vigor-organization-resilience-ecosystem services (VORS) model [2,19,22,23]. Among them, the VORS model has been the most widely used because it can fully reflect the inherent and complex characteristics of a regional ecosystem under the framework of human-earth system coupling, as well as the interaction between a natural ecosystem and the human socio-economic system [18,19,24]. However, it still has the following problems: first, in terms of setting the weight of resistance and resilience in the calculation formula of ecosystem resilience, previous studies have judged the overall economic development level and human activity intensity of the study area based on personal experience, and then assigned the weight of resistance and resilience, respectively [18,19,22]. However, in large-scale region studies, the economic development level and human activity intensity of different units within the study area have spatial heterogeneity, so the weights of resilience and resistance of different research units need to be assigned separately. Second, a few scholars have been begun to keep a watchful eye on spatial proximity effect for the past few years [19,22], but there are still two limitations. On the one hand, most studies only represent the service value of each ecosystem in the research unit through the service value coefficient of each ecosystem, without considering the difference of the total value of ecosystem services in each research unit. On the other hand, when calculating the spatial proximity effect coefficient, the existing research only considers the influence of the adjacent pixel on the four sides around the center pixel. However, the center pixel is not only affected by the four pixels adjacent to the edge, but also by the four pixels adjacent to the point. Therefore, the influence of eight adjacent pixels should be considered when calculating the spatial proximity effect coefficient. Based on this, the VORS model was improved by modifying the ecosystem elasticity formula and ecosystem service formula.

Since the 1990s, the importance of space for socioeconomic theory has been widely recognized, and a large number of theoretical and empirical studies have shown that spatial effects (spatial dependence and heterogeneity) have become common, rather than an exception in empirical studies [25]. Spatio-temporal interaction has become an important method and perspective for geographers to study environmental problems and socioeconomic sustainable development. At present, many scholars have discussed the effective integration of time factor into exploratory spatial data analysis (ESDA). The exploratory spatio-temporal data analysis (ESTDA) framework proposed by Rey implements the coupling and visual display of spatio-temporal association by introducing graph theory, which is a more beneficial exploration of spatio-temporal interaction analysis [25,26,27]. In recent years, with the implementation of ecological conservation strategies in the Yellow River Basin of China, and the increasing inter-regional ecosystem linkages and spatial spillover effects, it is possible that the ecosystem health levels among neighboring regions will appear agglomeration and similar dynamic patterns over time. In addition, based on the theories of ecosystem service flows and environmental externalities, the spatio-temporal behavior of ecosystem services and its eco-environmental effects flows within a region highlights the importance of spatio-temporal interactions in analyzing the dynamics of regional ecosystem health distribution. However, there is still a lack of studies on the spatio-temporal dynamics of regional ecosystem health from the perspective of spatio-temporal interaction, and most studies separate the interaction between spatial patterns and temporal processes.

As the main area for water and soil conservation and pollution prevention in the Yellow River Basin, the MRYR is an important ecological function area [11], as well as a typical climate-sensitive area and densely populated area. However, under the influence of climate and human activities, eco-environmental issues were very concentrated and prominent, such as soil erosion [6,11], land desertification [14,16,20], water and soil pollution [7,21], and vegetation degradation [16,28]. Ecosystem degradation had badly imperiled the ecosystem health of the MRYR and even the entire basin [19,20]. In view of this, based on the multi-source data, the improved VORS model was used to conduct a multi-scale assessment of ecosystem health in the MRYR, and then the ESTDA method was used to reveal the spatial and temporal interaction relationship and network characteristics of regional ecosystem health, in order to provide a reference for environmental management and regional sustainable development. This study proposed an improved assessment model of ecosystem health, explored the scale effect of ecosystem health assessment, and expanded the research on the spatio-temporal interaction characteristics of ecosystem health. The results of this study have important reference value for the current theory and application research of ecosystem health.

## 2. Materials and Methods

### 2.1. Materials

**Figure 1:**
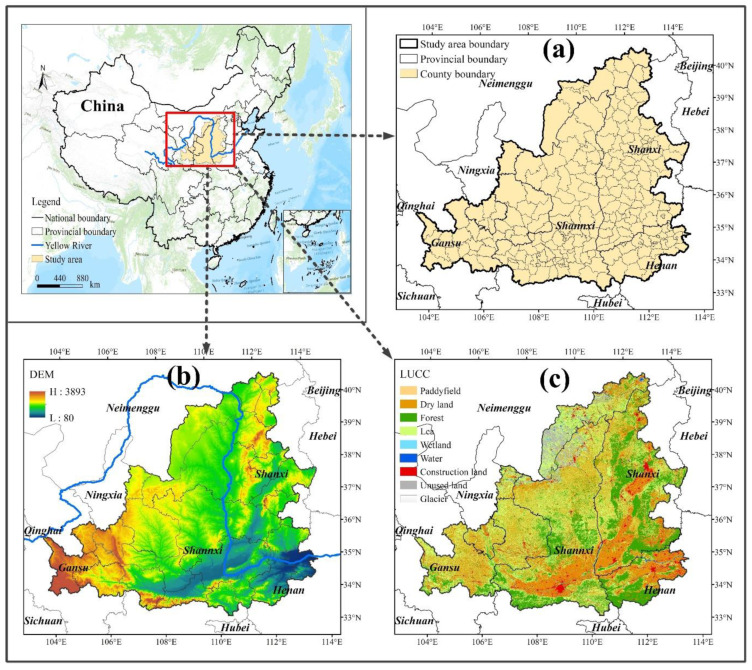
The Middle Reaches of the Yellow River (MRYR) in China, (**a**) Administrative division map of the MRYR, (**b**) Elevation distribution map of the MRYR, and (**c**) Land use distribution map of the MRYR in 2018.

(1) Study area. This study takes the Middle Reaches of the Yellow River (MRYR) as the study area, and the scope of the study area includes the 244 county administrative units involved in the Middle Reaches of the Yellow River (Figure 1). The MRYR starts from Hekou town in the west and ends at Huayuankou in the east. The geographical location was between 32° and 42° north and 104° and 113° east. The total area was about 36.3 × 10^4^ km^2^, accounting for 48.1% of the total area of the Yellow River Basin. The MRYR was not only the most typical climate sensitive area and important ecological function area in the Yellow River Basin, but also the main area where environmental problems such as ecological degradation occur. Therefore, the MRYR was the most typical case area for ecosystem health research.

(2) Data sources. In this study, the sources of administrative boundary, land use raster data, annual precipitation, annual average temperature, meteorological station data, net primary productivity, elevation, soil data, DMSP-OLS night light data are shown in Table 1. The normalized vegetation index (NDVI) is based on the Landsat MSS, Landsat TM/ETM and Landsat 8 series remote sensing images, and uses the Google Earth Engine (GEE) platform to synthesize monthly and annual NDVI data using Maximum Value Composite (MVC), including seven periods of normalized vegetation index raster data in 1990, 1995, 2000, 2005, 2010, 2015, and 2018, with a resolution of 250 m. The socioeconomic statistical data adopted in this paper were obtained from China urban statistical yearbook, Ningxia 60 years of glory (1958–2018), provincial statistical yearbook, and statistical bulletin of national socioeconomic development of counties. The data on the economic value of grain crops per unit comes from the China agricultural product price survey yearbook.

### 2.2. Methods

#### 2.2.1. Framework of Ecosystem Health Assessment

**Figure 2:**
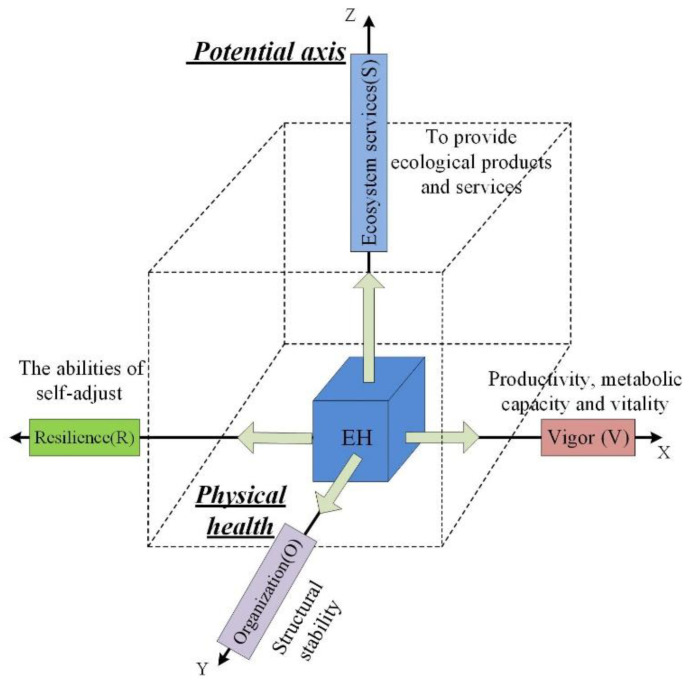
Framework of ecosystem health assessment.

The research object of regional ecosystem health is a “nature-economic-society” regional complex ecosystem that integrates natural ecosystems, human economic systems, and social systems [29]. Ecosystem health can be defined as that the regional composite ecosystem has the ability to remain its spatial structure and ecosystem process, self-regulation and self-renewal, and self-recovery in front of external stress, and can ensure the sustainable supply of ecosystem service function [18,30,31,32]. To systematically evaluate ecosystem health in ecologically fragile regions from the viewing angle of human-earth system coupling and sustainable development, this paper adopts the evaluation framework of “vigor—organization—resilience—ecosystem services” (VORS) to organize various subsystems and evaluation indicators based on the concept, intension, and research content of regional ecosystem health (Figure 2). The VORS framework not only considers ecosystem productivity, metabolic capacity and vitality level, the integrity and stabilization of ecosystem structure and functionality, and the elasticity to retain its structure and functionality, but also it reflects the sustainability of ecosystem services provided by ecosystems to humans, and strengthens the connection between natural ecosystems and human social system, so it can roundly and systematically represent the holistic health level of regional complex ecosystems [18,19,20,33]. In this framework (Figure 2), ecosystem vigor, organization, and resilience constitute the ontology health state plane of the regional ecosystem, and ecosystem services constitute the service function potential axis of the healthy development of the regional composite ecosystem. The ontology health state surface and service function potential axis are indispensable, and surface and axis together construct the holistic health level of regional composite ecosystem.

#### 2.2.2. Ecosystem Health Assessment Based on the Improved VORS Method

Considering the limitations of VORS model, this study introduced the spatial weight coefficient and the modified spatial proximity effect coefficient to improve the VORS model. The formula of the improved VORS model is expressed as Formulas (1) and (2):

(1) $$\mathrm{EHI}=\sqrt{\mathrm{PHI}\times \mathrm{ES}}$$

(2) $$\mathrm{PHI}=\sqrt[{3}]{\mathrm{EV}\times \mathrm{EO}\times \mathrm{ER}}$$

In the above formulas, EHI represents the ecosystem health level, PHI is the ecosystem ontology health level. EV is ecosystem vigor, EO is ecosystem organization, ER is ecosystem resilience, and ES represents ecosystem service value.

(1) Ecosystem vigor (EV). EV depicts the metabolic or primary prolificacy of regional ecosystem [2,19]. The NDVI is compactly made relevant to vegetation growth and net primary prolificacy, and has been broadly shown to be a valid index for evaluating ecosystem vigor level [2,19,22,34]. Hence, this study chose NDVI to represent the ecosystem vigor level.

(2) Ecosystem organization (EO). EO refers to the diversity of composite ecosystem elements composition and processes, i.e., the completeness and complicacy of regional ecosystem structure and functionality [29,34,35]. Ecosystem organization can be characterized by landscape heterogeneity (LH), holistic landscape connectivity (LC), and connectivity of important ecosystems (CIE) [2,22]. Among them, landscape heterogeneity is currently characterized by Shannon’s diversity index (SHDI) and area-weighted patch fractal dimension (AWMPFD). The landscape division index (DIVISION) and landscape contagion index (CONTAG) are usually used to characterize the holistic landscape connectivity [19,22,31]. The patch fragmentation index (PFN) and patch connectivity index (CON) of woodlands, grasslands, and watersheds are used to represent the important ecosystems connectivity [22]. The weights of LH, LC and CIE are assigned as 0.35, 0.35 and 0.3, severally, according to their relative importance [19,22,31,35,36]. The formula of ecosystem organization is expressed as Formula (3):

(3) $$\begin{array}{c} \mathrm{EO}=0.35\mathrm{LH}+0.35\mathrm{LC}+0.3\mathrm{CIE}\\ \;\;=\left(0.1\times \mathrm{AWMPFD}+0.25\times \mathrm{SHDI}\right)+\left(0.2\times \mathrm{DIVISION}+0.15\times CONTAG\right)+\\ \;\;\;\left(0.07\times PFN_{f}+0.03\times CON_{f}+0.07\times PFN_{w}+0.03\times CON_{w}+0.07\times PFN_{g}+0.03\times CON_{g}\right) \end{array}$$

In Formula (3), $PFN_{f},PFN_{g}$, and $PFN_{w}$ are the patch fragmentation indices of woodland, grassland, and water, respectively. $CON_{f},CON_{g}$, and $CON_{w}$ are the patch connectivity indices of forest, grassland, and water, respectively. To eliminate the possible differences in dimensions and magnitudes between indicators, we divide all indicators into positive indicators and reverse indicators before calculating organizational power, and then use the extreme value standardization method to standardize the data.

(3) Ecosystem resilience (ER). ER delegates to the capacity of a regional ecosystem to overcome internal and external pressures (resistance) during the stress process and the ability to restore its own structure and functionality (resilience) after the stress disappears [19,22,29]. Referring to the mature practice of pre-existing study [31], we use the resistance and resilience coefficient to measure the resistance value and resilience value (Table 2). Existing studies have judged the overall economic development level and human activity intensity of the study area based on personal experience, and then assigned the weights of resistance and resilience accordingly [19,22]. However, the level of economic development and the intension of human activity in different units within the study area are spatially heterogeneous, so it is necessary to spatially assign the weights of resilience and resistance to different study units. In view of this, this paper draws on the idea of piecewise function and introduces spatial weight coefficients to spatially correct the ecosystem elasticity formula. Among them, the subsection interval of the weight coefficient refers to the existing research results [2,22,31,34,35,36], and the ratio of the per capita *GDP* of the research unit to the per capita *GDP* of the research area is used as the division basis [37,38,39]. The revised formula of ecosystem resilience is expressed as Formulas (4)–(6):

(4) $${\mathrm{ER}}_{i}=w_{t}^{i}\times \sum_{m=1}^{9}P_{m}*Resil_{m}+\left(1-w_{t}^{i}\right)\times \sum_{m=1}^{9}P_{m}*Resist_{m}$$

(5) $$w_{t}^{i}=\left\{\begin{array}{c} 0.7\;,\;1.5<\frac{P\mathrm{er}GDP_{i}}{\bar{\;P\mathrm{er}GDP}}\;\\ 0.6\;,\;1.1<\frac{\;P\mathrm{er}GDP_{i}}{\bar{\;P\mathrm{er}GDP}}\leq 1.5\;\\ 0.5\;,\;0.9<\frac{\;P\mathrm{er}GDP_{i}}{\bar{\;P\mathrm{er}GDP}}\leq 1.1\;\\ 0.4\;,\;0.5<\frac{\;P\mathrm{er}GDP_{i}}{\bar{\;P\mathrm{er}GDP}}\leq 0.9\;\\ 0.3\;,\;0<\frac{\;P\mathrm{er}GDP_{i}}{\bar{\;P\mathrm{er}GDP}}\leq 0.5\; \end{array}\right.$$

(6) $$P\mathrm{er}GDP_{i}=\frac{GDP_{i}}{POP_{i}}$$

In Formulas (4)–(6), ${\mathrm{ER}}_{i}$ is the ecosystem resilience.$\;P_{m}$ is the ratio of the area of each landscape type to the total area of the study unit. $w_{t}^{i}$ is the ecosystem resilience weight, $1-w_{t}^{i}$ is the ecosystem resistance weight. $Resist_{m}$ is the resistance coefficient, $Resil_{m}$ is the resilience coefficient (Table 2). $P\mathrm{er}GDP_{i}$ is the per capita *GDP*, $\bar{\;P\mathrm{er}GDP}$ is the average of per capita *GDP* of all research units in the same period, $GDP_{i}$ is the total *GDP*, $POP_{i}$ is the total population.

(4) Ecosystem services (ES). ES delegates to the life-sustaining products and services acquired immediately or mediately through the structure, process, and functionality of the ecosystem. It is compactly connected with human health and well-being [40,41,42]. The continuous feed power of ecosystem service functions and the analysis of the cost-benefit of ecosystem services are the key criteria to achieving the overall and macroscopic assessment of ecosystem health under the framework of human-land coupling, reflecting the interaction between natural ecosystems and human socioeconomic systems [18,19]. From the perspective of ecosystem service flow, ecosystem services can be delivered to spatially adjacent regions [18,43]. Therefore, we should consider the spatial proximity interaction effect when calculating ecosystem services, and further revise the assessment results of ecosystem services. Calculation steps of ecosystem service value based on spatial proximity interaction effect: (1) Compute the amount of ecosystem service value of the standard equivalent factor of each research unit (Formula (7)). (2) According to the three temporal and spatial dynamic factors of net primary productivity of vegetation (NPP), precipitation and soil retention, correct the basic equivalent table of ecosystem service value (Table S1), and then use the equivalent factor method to compute the total value of ecosystem services in each year and each research unit (Formula (8)). (3) Compute the spatial proximity total effect coefficient of each study unit (Formula (9)). (4) Calculation of the ecosystem service value based on the spatial proximity effect (Formula (9)). The revised formula of ecosystem service is expressed as Formulas (7)–(9):

(7) $$D_{i}=1/7\times \left[\frac{1}{A_{i}}\sum_{k=1}^{l}\left(S_{ki}\times V_{ki}\right)\right]$$

(8) $$F_{nij}=\left\{\begin{array}{c} N_{ij}\times F_{n1}\\ R_{ij}\times F_{n2}\\ S_{ij}\times F_{n3} \end{array}\right.$$

(9) $$\begin{array}{c} ESV_{ij}\left(\mathrm{SNE}\right)=ESV_{ij}\times C{\mathrm{SNE}}_{ij}\\ =\overset{10}{\underset{a=1}{\sum }}\left(D_{ij}\times F_{ij}^{a}\times LA_{ij}^{a}\right)\times \left[\frac{\sum_{k=1}^{m}\left(1+\frac{\sum_{c=1}^{8}S_{k}^{c}}{100}\right)}{m}\right]\\ =\sum_{a=1}^{10}\left(D_{ij}\times F_{ij}^{a}\times LA_{ij}^{a}\right)\times \left[\frac{\sum_{k=1}^{m}\left(1+\frac{\left(S_{k}^{1}+S_{k}^{2}+S_{k}^{3}+S_{k}^{4}+S_{k}^{5}+S_{k}^{6}+S_{k}^{7}+S_{k}^{8}\right)}{100}\right)}{m}\right] \end{array}$$

In Formulas (7)–(9), $D_{i}$ is the ecosystem service value of the standard equivalent factor of the *i*th research unit. $S_{ki}$ is the yield (kg) of the ith research unit and the kth food crop (three food crops, rice, wheat, and corn are selected). $V_{ki}$ is the unit food crop of the kth food crop economic value (yuan/kg). $A_{i}$ are the total sown area (ha) of food crops. *n*1 represents the service functions of food production, raw material production, gas regulation, climate regulation, environmental purification, maintenance of nutrient cycle, biodiversity, and aesthetic landscape, n2 represents the service function of water resources supply and hydrological regulation, and n3 represents the service function of soil conservation. $N_{ij}$ is the spatiotemporal dynamic correction factor of NPP, $R_{ij}$ is the temporal and spatial dynamic correction factor of precipitation, $S_{ij}$ is the temporal and spatial dynamic correction factor of soil conservation. Revised universal soil loss equation (RUSLE) was adopted to compute the soil retention [44].$\;ESV_{ij}$ is the total value of ecosystem services,$\;LA_{ij}^{a}$ is the area of land type *a*. $ESV_{ij}\left(\mathrm{SNE}\right)$ is the ecosystem service value based on the spatial proximity effect, $C{\mathrm{SNE}}_{ij}$ is the total effect coefficient of spatial proximity. $S_{k}^{c}$ is the spatial proximity effect coefficient of ecosystem services corresponding to the *c*-th pixel around the central pixel *k* (Table 3). *m* is the number of pixels included in research unit *i*. It should be noted that referring to the previous study results [18,19,22], we combined desert and bare land into unused land for unified calculation in the process of calculating the total effect coefficient of spatial proximity.

#### 2.2.3. Optimal Analysis Scale Selection Based on Semi-Variogram

In this study, grid units were used as basic research units for ecosystem health assessment and influencing factor analysis. In the selection of grid cell size (analysis scale), due to the spatial variability of landscape pattern index at different analysis scales, it is necessary to determine the optimal analysis scale before calculating landscape index. In this study, semi-variogram was used to determine the optimal analytical scale. The semi-variogram is a function that takes the size of statistical correlation coefficient as a distance, specifically including five important parameters: Range, Nugget, denoted C0. Partial Still value, denoted as C. Sill value, denoted as C+CO. The ratio of Nugget value to Sill value is the Nugget-Sill ratio, denoting CO/(C+CO). According to previous research experience [45,46], when the Nugget-Sill ratio reaches a stable state, it indicates that the spatial variation of landscape index tends to be stable, and the scale at this time can be judged as the characteristic scale of the research region.

#### 2.2.4. Variation Coefficient, Theil Index and Theil Decomposition Index

The variation coefficient (CV) and Theil index (Theil) can reflect the degree of dispersion of the data set, and the level of regional differences [47,48]. In our study, the CV and Theil are adopted to estimate the regional disparity of ecosystem health. The model formula is expressed as Formulas (10) and (11):

(10) $$\mathrm{CV}\;\;\frac{1}{\bar{y}}\sqrt{\frac{\sum_{i=1}^{n}{\left(y_{i}-\bar{y}\right)}^{2}}{n}}$$

(11) $$\mathrm{Theil}=\frac{1}{n}\sum_{i=1}^{n}\frac{y_{i}}{\bar{y}}log\left(\frac{y_{i}}{\bar{y}}\right)$$

In Formulas (10) and (11), *n* is the number of samples, $y_{i}$ is the ecosystem health level, and $\bar{y}$ is the average value of ecosystem health.

Theil decomposition index can decompose the overall disparity into intra-regional and inter-regional disparity. To prevent the difference from being offset when the Theil index is negative, this paper uses the improved Theil decomposition index to analyze the regional disparity. The model formula is as follows [47,49]:

(12) $$\begin{array}{c} \mathrm{Theil}=\sum_{i=1}^{n}R_{i}\ln\left(nR_{i}\right)=T_{W}+T_{B} \end{array}$$

(13) $$T_{W}=\sum_{k=1}^{K}R_{k}\left|\underset{i\in g_{k}}{\sum }\frac{R_{i}}{R_{k}}\log\left(\frac{R_{i}/R_{k}}{1/n_{k}}\right)\right|$$

(14) $$T_{B}=\sum_{k=1}^{K}R_{k}\left|\log\left(R_{k}\frac{n}{n_{k}}\right)\right|$$

In Formulas (12)–(14), $T_{W}$ is the intra-regional disparity. $T_{B}$ is the inter-regional disparity. *k* is the number of regions divided, i.e., including the east, middle and west. $n_{k}$ is the samples number of group *k*. $R_{i}$ is the proportion of the ecosystem health value. $R_{k}$ is the proportion of the ecosystem health value of the *k*-th group.

#### 2.2.5. Exploratory Space–Time Data Analysis Method

The exploratory space–time data analysis (ESTDA) method can achieve coupling of temporal and spatial associations and clarify the spatiotemporal interaction characteristics of evaluation units. The ESTDA method mainly includes the LISA time path, LISA space–time transition, and spatio-temporal network analysis [25,27].

(1) LISA time path. The LISA time path is the temporal continuity expression of position transfer of spatial units in local spatial autocorrelation Moran’s I scatter diagram [25,27,50]. Through the visual space unit attribute value and its neighborhood average space (lag), pairs move and can reflect the degree of spatial and temporal interaction between space unit, direction, competition situation, and the influence of time and space depend on the effect of regional system evolution. In this study, the LISA time path indexes of each space unit include relative length (Γ) and curvature (ε), and the specific calculation formulas are expressed as Formulas (15)–(17):

(15) $$\Gamma_{i}=\frac{\sum_{t=1}^{T-1}d\left(L_{i,t},L_{i,t+1}\right)}{\left[\sum_{i=1}^{n}\sum_{t=1}^{T-1}d\left(L_{i,t},L_{i,t+1}\right)\right]/n}$$

(16) $$\varepsilon_{i}=\frac{\sum_{t=1}^{T-1}d\left(L_{i,t},L_{i,t+1}\right)}{d\left(L_{i,1},L_{t,T}\right)}$$

(17) $$L_{i,t,}=\frac{z_{i,t}\times \sum_{j}\left(w_{i,j}\cdot z_{j,t}\right)}{\sum_{i}z_{i,t}^{2}}$$

In the formula, $\Gamma_{i}$ is the relative length of movement of the ith space unit, $\varepsilon_{i}$ is the curvature of the movemen, $d\left(L_{i,t},L_{i,t+1}\right)$ is the moving distance between *t* and *t* + 1 years, $L_{i,t}$ is the coordinates of Moran’s *I* scatterplot of the *t* year. $z_{i,t}$ is the z-normalized ecosystem health value. $w_{i,j}$ is the spatial adjacency weight. The longer $\Gamma_{i}$, the more dynamic local space dependence and local space structure. The larger the $\varepsilon_{i}$ is, the more dynamic the local space dependent direction and the more fluctuating development process.

(2) LISA space–time transition. LISA provided a decomposed perspective to reveal the local spatial dependence [51,52]. Based on this, Rey proposed the local Markov transition and LISA space–time transition [25,27,50]. Specifically, it can be divided into four types of space–time transitions: Type I represents the type in which neither the spatial unit itself nor its neighbors changes in spatial form. Type II represents only the spatial unit itself. In a transition, its neighborhood does not change. Type III means that only the neighborhood of the space unit transitions. Type IV means that both the space unit and its neighborhood transition, and this type can be further split into IV(1) and IV(2); IV(1) means that the transition direction of the space unit and its neighbor unit is the same, IV(2) means that the transition direction of the space unit and its neighbor unit are opposites. Furthermore, the four types can be divided into either a space–time transition (SF) and space–time cohesion probability (SC). Its formula is expressed as Formulas (18) and (19):

(18) $$\mathrm{SF}=\frac{F_{2}+F_{3}}{m}$$

(19) $$\mathrm{SC}=\frac{F_{1}+F_{4A}}{m}$$

In the formula, *m* is the total sum of transitions in a time period. SF is the spatiotemporal transition probability; SC is the spatiotemporal condensation probability. $F_{1},F_{2},F_{3}$ and $F_{4A}$ represent the transition numbers of type I, type II, type III, and type IV(1), respectively.

(3) Time-space interactive visualization. The ESTDA method can explicitly express the spatiotemporal interaction pattern of regional complex systems through graph theory [50]. The spatiotemporal network of ecosystem health distribution can be spatially visualized through the central connection of spatial units, so as to reveal the similarity between the competition and cooperation situation and development mechanism between spatial units [25]. The spatiotemporal network flow of unit interaction in the regional system can be visually expressed by calculating the covariance correlation coefficient of the time moving trajectory of the adjacent unit Lisa.

## 3. Results

### 3.1. Optimal Analysis Scale of Ecosystem Health

**Figure 3:**
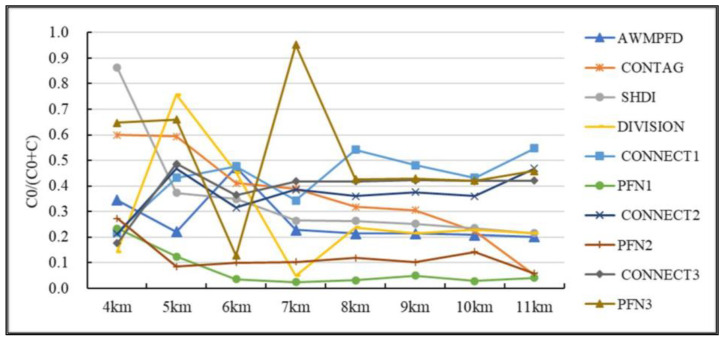
Characteristic values of spatial variation of landscape pattern indexs at different analytical scales.

Landscape indicators were used as the analysis indexes of the optimal analysis scale. The year selected for semi-variogram analysis was the middle year of the study period, namely 2005, and the analysis scale selected included distances of 4 km, 5 km, 6 km, 7 km, 8 km, 9 km, 10 km, and 11 km. An analysis of different scales of landscape pattern index block base than changes the trends (Figure 3); the Nugget-Sill ratio reaches a stable state at 8 km to 10 km, which indicates that the spatial variation of landscape pattern index at this analysis scale tends to be stable, and the scale at this time can be determined to be the optimal analysis scale (characteristic scale). In addition, a large scale will cause more spatial information loss. Therefore, the optimal analysis scale selected in this study is 8 km × 8 km, with a total of 6600 grid units.

### 3.2. Spatio-Temporal Characteristics of Ecosystem Health

#### 3.2.1. Time Series Evolution

In this study, maximum, minimum, median, mean, upper and lower quartiles, and outliers were used to analyze the time series change characteristics of ecosystem health in the MRYR. Furthermore, the variation coefficient, Theil index and Theil decomposition index were used to analyze the temporal variation characteristics of regional differences in ecosystem health.

**Figure 4:**
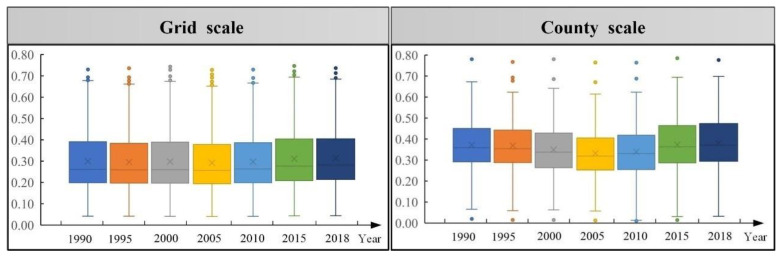
Box plot of ecosystem health change at grid and county scale.

As shown in Figure 4, in terms of maximum value and minimum value, the ecosystem health level at grid and county scale decreased first and then increased. In the study period, the median and mean values showed a synchronous change in decreasing first and then increasing, indicating that the ecosystem health level in the middle reaches of the Yellow River generally showed a trend of decreasing first and then increasing. In terms of the distance between the upper and lower quartiles, the values of the top 25% research units tend to be concentrated, showing the convergence phenomenon of high-value clubs. The lower quartile distance showed an increasing trend of fluctuation, indicating that the values of the last 25% study units tended to be dispersed.

**Figure 5:**
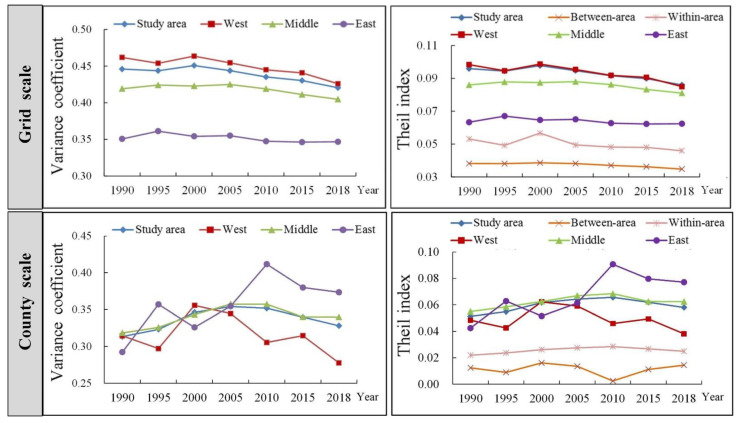
Variation trends of the variance coefficient and Theil index of ecosystem health at grid and county scale.

From the perspective of regional differences (Figure 5), there were large regional differences of ecosystem health in the study area, eastern, central, and western regions from 1990 to 2018. In terms of time series, the regional differences between the study area and the eastern, central, and western regions increased first and then decreased. Furthermore, Theil decomposition index was used to decompose the overall differences in the study area into the intra-group and inter-group differences in the eastern, western, and central regions. The intra-group differences in the eastern, central, and western regions increased first and then decreased, while the inter-group differences showed differences at the grid and county scales. The temporal changes in inter-group differences at the grid scale were more stable than those at the county scale.

#### 3.2.2. Spatial Pattern Evolution

According to the data distribution of ecosystem health index (EHI), the study region was divided into five areas. The dividing standard is Low level area (0 < EHI ≤ 0.25), Lower level area (0.25 < EHI ≤ 0.4), Middle level area (0.4 < EHI ≤ 0.5), Higher level area (0.5 < EHI ≤ 0.6), High level area (0.6 < EHI).

**Figure 6:**
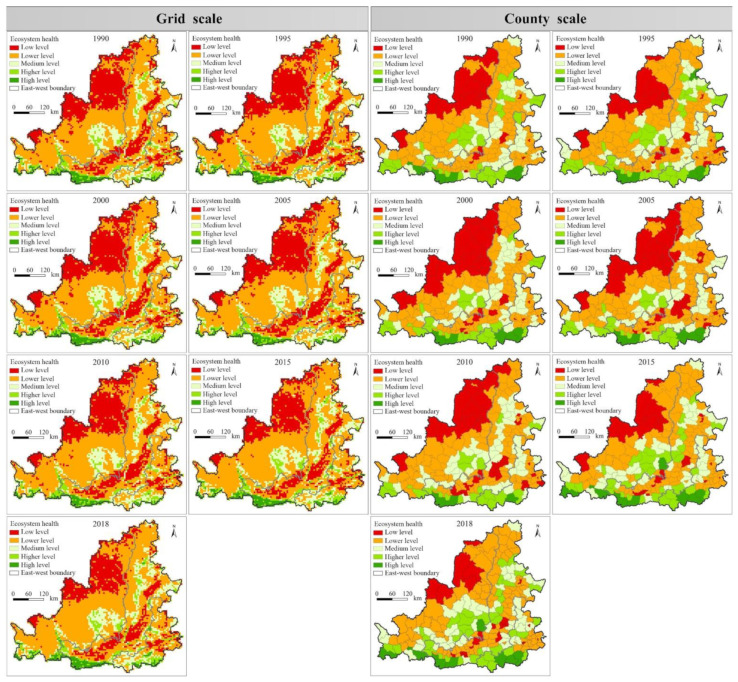
Spatial evolution of ecosystems health level in the MRYR from 1990 to 2018.

From the perspective of spatial distribution, the ecosystem health level in the MRYR from 1990 to 2018 showed the hierarchical structure characteristics of alternating distribution of low-value zone and high-value zone (Figure 6). In detail, the high level area was primarily distributed in the mountainous and hilly areas in the south of the study area, including the southwest of Liupan Mountain, Qinling Mountain, southeast of Gushan Mountain, and west of Funiu Mountain. These areas have superior natural endowment conditions. The higher and medium level areas are primarily distributed in the Northern Shaanxi plateau, Fenhe River Basin, Luliang Mountain Area, and south of Taihang Mountain. Among them, the health level of northern Shaanxi Plateau has been improved significantly since 2010. The low level areas and lower level areas are mainly distributed in the south of Kubuqi Desert, the East and south of Mu Us sandy land, Yinchuan City, Guanzhong Basin with dense population and cities, Luohe River Basin, Fenhe River Basin, and the west of North China Plain. In general, the spatial evolution of grid and county scale ecosystem health in the middle reaches of the Yellow River was characterized by spatial imbalance and time non-stationarity. The spatial evolution of ecosystem health in the rapid urbanization areas in the central and eastern regions and the fragile ecological areas in the central and western regions showed more dynamic local spatial dependence and local spatial structure. From the comparison of the two research scales, the grid scale can describe the spatial distribution characteristics of ecosystem health more refined, indicating the existence of spatial scale effects in ecosystem health assessment.

### 3.3. Spatio-Temporal Interaction Characteristics of Ecosystem Health

#### 3.3.1. LISA Time Path Analysis

**Figure 7:**
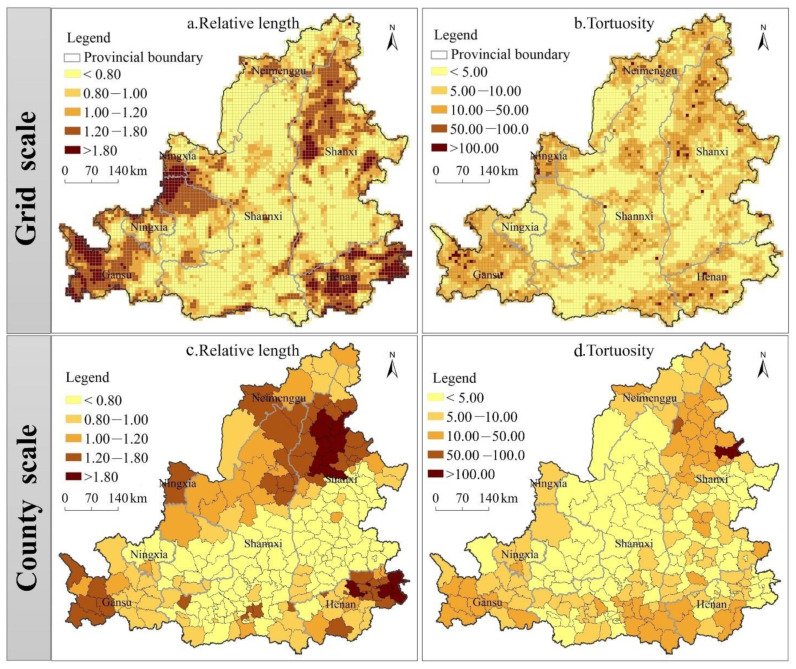
Spatial distribution of relative lengths and tortuosity of LISA temporal paths.

According to the relative length and tortuosity, the temporal evolution path of the ecosystem health local space in the MRYR during the study period can be analyzed. In terms of the relative length (Figure 7a,c), the research units with a relative length greater than 1 are primarily distributed in the rapidly urbanized regions and the ecologically fragile regions in the midwest regions, including the border area of Shaanxi, Gansu and Ningxia provinces, Ordos Municipal District, eastern area of the Mu Us Desert, southern Gansu, central area of the Northern Shaanxi Plateau, Guanzhong Basin, Taiyuan city circle, Jinzhong municipal district, western North China Plain, West Henan Mountain Area, indicating that these regions have a more dynamic local spatial dependence and local spatial structure. In terms of the curvature of the LISA time path (Figure 7b,d), the curvatures from 1990 to 2018 were all greater than 1, the curvature generally shows a spatial distribution characteristic of high in the east and low in the west, high in the south-north and low in the middle. The regions with large tortuosity are primarily distributed in the Taiyuan urban circle, the Linfen-Changzhi-Jincheng urban belt, the Ordos municipal area, the southern of Gansu Province, the Guanzhong Basin, the southern of Shaanxi Province, the mountainous areas of western Henan, rapidly urbanized areas, and the transitional belt of mountainous and plains, indicating that the ecosystem health has a dynamic local space-dependent direction and a strong spatio-temporal dependence effect. In general, the rapid urbanization areas, the ecologically fragile areas in the central and western regions, and the transitional zone of mountainous and plain have more dynamic local spatial structure, and more complex process of spatio-temporal interaction, while the other regions have strong path dependence and lock-in characteristics. Compared with county scale, the spatial heterogeneity of relative length and curvature of grid scale was more obvious, so it shows obvious spatial scale effect.

#### 3.3.2. LISA Space-Time Transition Analysis

The LISA space–time transition analysis method can further depict the transition of the Local Moran’s I scatter plot between different spatial forms. As can be seen in Table 4 and Table 5, The spatio-temporal condensation probability at the grid and county scales has remained above 0.75, indicating that most regions have strong path-dependent and lock-in characteristics. The periods of 1990–1995, 1995–2000, 2000–2005 and 2005–2010 were in the stage of strong interaction, and 2010–2018 was in the stage of weak interaction, indicating that the spatial correlation structure of the regional system tended to be stable after 2010.

**Figure 8:**
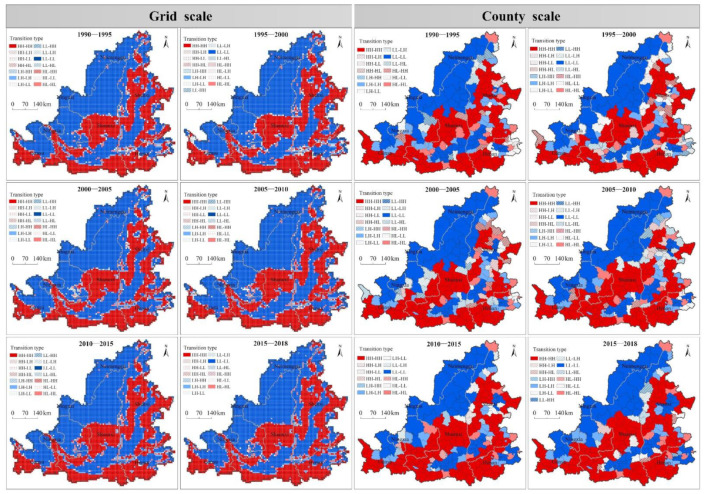
Ecosystem health Local Moran’s I spatial transition map at grid and county scale.

From the local spatial migration characteristics (Figure 8): (1) Local spatial heterogeneity was strong. The regions where the spatio-temporal transitions occurred were mainly distributed in the junction of the northwest low-value belt and the central high-value belt, Hohhot and Ulanqab city, Taiyuan urban agglomeration, Xi’an urban agglomeration, Zhengzhou—Luoyang urban agglomeration, and the surrounding areas of the Northern Shaanxi Plateau. (2) The characteristics of stage changes were significant. It is mainly manifested in two stages: 1990–2010 and 2010–2018. From 1990 to 2010, the regions with strong spatial interaction are mainly distributed in Yuncheng city, Sanmenxia city, the surrounding area of Taiyuan city, the northern of Shanxi Province, the surrounding area of Northern Shaanxi Plateau, Baoji city and Zhengzhou-Luoyang urban agglomeration. From 2010 to 2018, the areas with strong spatial interaction decreased significantly, primarily distributed in the periphery of the Northern Shaanxi Plateau, Fenhe River Basin, Taiyuan city circle, and Xi’an metropolitan area. (3) The boundary between the northwest low-value belt and the central high-value belt, the central part of the Northern Shaanxi Plateau and the rapidly urbanization area were dominated by spatial spillover effect, while the transitional zone between plain and mountain area was dominated by the spatial polarization effect. In the semi-arid region of northwest China, the overall health level of regional ecosystem was relatively low, under the influence of negative environmental externalities, the negative spatial spillover effect of regional system to the boundary area was much higher than the spatial polarization effect. In rapid urbanization areas such as Taiyuan city circle, Xi’an city circle and Zhengzhou city circle, the negative spatial spillover effect caused by urban expansion and economic growth on ecosystem health was much higher than the spatial polarization effect. Affected by the positive environmental externality brought by the improvement of ecological environment quality, the positive spatial spillover effect in the central part of the Northern Shaanxi plateau was much higher than the spatial polarization effect as a whole.

#### 3.3.3. Spatio-Temporal Networks of Ecosystem Health Interactions

**Figure 9:**
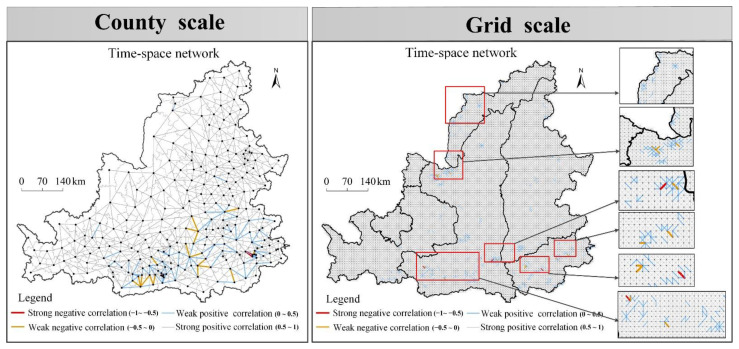
Spatio-temporal network of ecosystem health interaction in the MRYR.

The spatio-temporal network of ecosystem health interaction can describe the interaction characteristics of regional systems in detail and reveal the geographical nature behind ecosystem health. From the grid scale (Figure 9), there were 51186 pairs of positive correlation combinations, accounting for 99.94%, indicating that the ecosystem health in the MRYR mainly increased or decreased cooperatively during the evolution process. There were 30 pairs of negative correlation combinations, accounting for 0.06%, which were mainly distributed in Ordos city, south area of Mu Us Desert, central area of the Northern Shaanxi Plateau, Guanzhong Plain, southern Liupan Mountain area, Yan’an city and Weinan city, Weinan city and Yuncheng city along the Yellow River, Fenhe River basin, Western Henan Mountain area, and Zhengzhou-Luoyang City belt. At the county scale (Figure 9), there were 1266 pairs of positive correlation combinations, accounting for 95.91%, indicating that the county ecosystem health mainly increased or decreased cooperatively during the evolution process. There were 54 pairs of negative associations, accounting for 4.1%, mainly distributed in Guanzhong plain, Yan’an City and Weinan city, Linfen city of Shanxi Province, Fenhe River basin, and Luoyang city.

In total, the regional system is mainly growing or decreasing cooperatively in the evolution process. There was strong spatio-temporal competition in the process of time evolution in six typical regions, such as the sur-rounding areas of provincial capitals, the fringe areas of cities, the transitional zone between mountainous and basin in the central and western regions, the transitional zone of ecologically fragile regions, the mountainous areas of western Henan Province, and the areas along rivers. In addition, although the rapid urbanization area of the mideastern region (economically developed cities and economic underdeveloped city), the transition zone of eastern mountains and plain (mountainous city and plain city), marginal areas of the Loess Plateau (ecological restoration and its positive environmental externality effects) have not shown negative correlation in the study period, but they have shown weak positive correlation, i.e., they gradually tend to have spatio-temporal competition. In the process of time evolution, the relationship between adjacent units in this kind of region gradually evolves into a form of spatio-temporal competition, which reveals the non-cooperative characteristics of ecosystem health. Therefore, the problem of regional coordinated development should also be considered in the future development process.

## 4. Discussion

### 4.1. The Improved VORS Method

Based on the VORS model, this study introduced the spatial weight coefficient and the modified spatial proximity effect coefficient to modify the ecosystem resilience and ecosystem service value, respectively, so as to realize the improvement of VORS model and make the ecosystem health assessment results more scientific. This is the first contribution of this study. However, the complexity and comprehensiveness of regional composite ecosystems determine the complexity of research methods and the means to evaluate the health status of regional composite ecosystem. The evaluation indicators and evaluation models of ecosystem health are still in a stage of rapid development, and there are still many areas to be explored. In the future, we can explore new evaluation indicators and evaluation models by combining multidisciplinary models, RS and GIS technologies, providing new ideas and methods for existing research, and providing more accurate and comprehensive research results for future regional ecosystem health research.

### 4.2. The Analytical Scale

The analytical scale (spatial resolution) of regional ecosystem health mainly includes macro and meso scale perspectives. Among them, the macro scale includes provinces [53,54], cities [19,31,32,33], and township. The mesoscale includes grid units [2,20,36], protected areas and lakes. From the perspective of research objects and objectives, the macro and meso scale research focuses on the overall sustainability of regional ecosystems, mainly involving geography and environmental science.

In this paper, we conducted multi-scale assessment of ecosystem health at county and grid scales, and explored the scale effects of ecosystem health assessment. The results show that the grid scale can describe the spatial distribution characteristics and spatio-temporal interaction characteristics of ecosystem health in a more detailed manner, so it can avoid the loss of micro-information and facilitate the detailed observation of ecological risks. Although there is loss of micro-information in the research results at the county scale, an assessment based on county scale is beneficial to ecological management zoning and regulation. Therefore, the combination of grid scale and county scale research can produce the role of complementary advantages. In addition, existing studies mostly set the resolution of grid cells based on subjective experience, without considering the spatial variability of different analysis scales, and without using quantitative methods to determine the optimal analysis scale. In this study, considering the spatial variability of the analysis scale, it is a beneficial attempt to quantitatively determine the optimal analysis scale using the semivariogram, which makes the ecosystem health assessment results more scientific and the spatial information more robust to a certain extent.

### 4.3. Spatio-Temporal Interactions Networks

From the points of view of environmental externality theory and ecosystem service flow theory, climate, topography, hydrological processes, the fragile ecological environment, and human activity shaped the low-value aggregation state of ecosystem health and the synergistic relationship between adjacent units in the western semi-arid region of the MRYR, and promoted the convergence and spatial spillover of the regional system to a certain extent. From the point of view of urban agglomeration theory and growth pole theory, population and resources to the highly urbanization region agglomeration caused the rapid expansion of the town, high resource consumption, environmental pollution, and ecological service function of supply geographical spatial imbalance, triggering neighborhood units (the provincial capital city and its surrounding cities, the edge of the city) uncoordinated development, and further limits to the convergence of regional ecosystem health. The transition zone of mountainous and basin (Guanzhong Basin and Qinling Mountain, Guanzhong Basin, and Liupan Mountain), West Henan Mountain Area, along the Yellow River area (Luohe River and Fenhe River), and the ecologically fragile zone transition zone (the transitional area between the southern Mu Us Sandy Land and the Northern Shaanxi Plateau) are all regions with strong or weak negative correlation. It indicates that there is strong spatio-temporal competition between adjacent units in the above regions.

In regions that tend to compete with space and time (the rapid urbanization area of mideastern region, the transitional zone of mountains and plains in the eastern and marginal areas of the Loess Plateau). In the process of time evolution, the relationship between adjacent units in this kind of region gradually evolves into a form of spatio-temporal competition, which reveals the non-cooperative characteristics of ecosystem health. Therefore, the problem of regional coordinated development should also be considered in the future development process.

### 4.4. Future Research Direction

Until now, studies on ecosystem health evaluation and influencing factors have been mature, but the studies on the influencing mechanism and multi-scenario simulation of ecosystem health are still weak, and some problems still need to be further studied. To be specific: (1) Further explore the influencing mechanism of climate and human activities on ecosystem health. Although this study systematically analyzed the spatiotemporal impacts of climate and human activities on ecosystem health, the mechanism of the impacts has not been fully revealed. Therefore, the spatial-temporal impact process and interaction mechanism of climate and human activities on regional ecosystem health are still the focus and difficulty of future research. (2) Multi-scenario simulation of ecosystem health. Because it is difficult to obtain the auxiliary data of future scenarios required for the spatial simulation of ecosystem health, how to accurately simulate the spatial distribution of regional ecosystem health under the combined influence of future climate and human activities is still a challenge. In future research, multivariate data and a variety of model methods should be used to carry out long-term, high-precision, and multi-scenarios of ecosystem health spatial simulation research. (3) It is a momentous scientific and practical question to put forward a multi-scale ecological management zoning method and targeted ecological regulation strategy based on ecosystem health.

## 5. Conclusions

In the face of the lack of research on ecosystem health assessment methods, scale effects and spatio-temporal interaction characteristics, this study introduced the improved VORS model to carry out multi-scale assessment of ecosystem health in the MRYR to explore the spatio-temporal interaction characteristics of regional ecosystem health, which will be of great significance for the development of ecosystem health theories and environmental management. The main conclusions are as follows:

(1) From 1990 to 2018, the ecosystem health level at grid and county scale in the MRYR showed a trend of first decline and then increase, and experienced a slow decline and a steady rise from 1990 to 2005 and 2005 to 2018, respectively. The regional difference of ecosystem health was large, and it increased first and then decreased in time series. In terms of spatial pattern, the ecosystem health level at grid and county scale presented a spatially hierarchical structure with alternating low-value and high-value zones.
(2) In terms of scale effects of ecosystem health, the inter-group differences in the eastern, central, and western regions showed differences at grid and county scales, i.e., the temporal change in inter-group differences at grid scale was relatively stable compared with that at county scale. Compared with the county scale, the grid scale can describe the spatial distribution characteristics of ecosystem health more refined, indicating the existence of spatial scale effects in ecosystem health assessment.
(3) In terms of spatio-temporal interaction characteristics, the period from 1990 to 2010 was in the strong interaction period, and the period from 2010 to 2018 was in the weak interaction period. The rapid urbanization areas, the ecologically fragile areas in the central and western regions and the transitional zone between mountain and basin have more dynamic spatial structure and stronger spatio-temporal interaction process. The boundary between the northwest low-value zone and the central high-value zone, the central part of the northern Shaanxi Plateau and the rapid urbanization area are dominated by the spatial spillover effect, while the transitional zone between plain and mountain area is dominated by the spatial polarization effect.
(4) In terms of spatio-temporal interaction network, the regional system is mainly growing or decreasing cooperatively in the evolution process. Locally, there were strong spatio-temporal competition in the process of time evolution in six typical regions, such as the surrounding areas of provincial capitals, the fringe areas of cities, the transitional zone between mountainous and basin in the central and western regions, the transitional zone of ecologically fragile regions, the mountainous areas of western Henan Province, and the areas along rivers. In addition, the rapid urbanization areas in the mid-east region, the transitional zone between mountains and plains in the east region, and the marginal zone of the Loess Plateau gradually tend to exhibit spatio-temporal competition.

## Figures and Tables

**Table 1:** Data sources in the study.

Serial Number	Variable Name	Data Type/Resolution	Sources
1	Land use data	Raster data/30 m	http://www.resdc.cn, (accessed on 8 October 2022).
2	Normalized difference vegetation index	Raster data/250 m	http://www.resdc.cn, (accessed on 8 October 2022).
3	Annual precipitation	Raster data/1 km	http://www.geodata.cn, (accessed on 8 October 2022).
4	Annual average temperature	Raster data/1 km	http://www.geodata.cn, (accessed on 8 October 2022).
5	Meteorological station data	-	http://data.cma.cn, (accessed on 8 October 2022).
6	Net primary productivity	Raster data/5 km	http://www.geodata.cn, (accessed on 8 October 2022).
7	Elevation	Raster data/500 m	http://www.resdc.cn, (accessed on 8 October 2022).
8	Soil data	Raster data/30 m	http://westdc.westgis.ac.cn, (accessed on 8 October 2022).

**Table 2:** Resistance and resilience coefficients.

Landscape Type	Paddy Field	Dry Land	Woodland	Grassland	Wetlands	Water	Construction Land	Unused	Glacier
Resistance	0.6	0.5	1	0.7	0.6	0.8	0.3	0.2	0.1
Resilience	0.3	0.4	0.6	0.8	0.7	0.7	0.2	0.1	0.1

**Table 3:** Spatial proximity effect coefficients of ecosystem services for each land use type.

Land Use Type of Adjacent Pixel	Land Use Type of Center Pixel								
	Woodland	Grassland	Water	Paddy Field	Dry Land	Unused	Construction Land	Wetlands	Glacier
Woodland	+5	+5	+5	+5	+4	+4	+4	+5	+3
Grassland	+4	+5	+4	+2	+2	+3	+3	+4	+2
Grassland	+5	+4	+5	+4	+2	+4	+4	+4	+1
Paddy field	−1	−1	−5	+4	+2	−1	+1	−2	−1
Dry land	−1	−1	−4	+1	+2	−1	+1	−3	−1
unused	−1	−2	+5	−3	−3	+1	−1	−3	−1
Construction land	−2	−3	−5	+2	+2	−2	+1	−4	−3
Wetlands	+4	+3	+5	+3	+2	+3	+3	+5	+2
glacier	−4	−1	−2	−5	−5	−1	−1	−2	+1

**Table 4:** Local Moran’s I transition matrix and spatiotemporal transition of ecosystem health at grid scale.

**1990–1995**	***t*/*t* + 1**	**HH**	**LH**	**LL**	**HL**	**Transition Type**	**Amount**	**Proportion**	**SF**	**SC**
	HH	2388	31	3	37	I	6410	0.971	0.028	0.972
	LH	11	306	41	0	II	79	0.012		
	LL	4	19	3502	14	III	104	0.016		
	HL	7	0	23	214	IV	7	0.001		
**1995–2000**	***t*/*t* + 1**	**HH**	**LH**	**LL**	**HL**	**Transition Type**	**Amount**	**Proportion**	**SF**	**SC**
	HH	2367	21	11	11	I	6348	0.962	0.036	0.964
	LH	39	289	28	0	II	104	0.016		
	LL	3	53	3492	21	III	133	0.020		
	HL	41	1	23	200	IV	15	0.002		
**2000–2005**	***t*/*t* + 1**	**HH**	**LH**	**LL**	**HL**	**Transition Type**	**Amount**	**Proportion**	**SF**	**SC**
	HH	2389	26	5	30	I	6380	0.967	0.031	0.969
	LH	26	303	35	0	II	106	0.016		
	LL	8	27	3485	34	III	101	0.015		
	HL	9	0	20	203	IV	13	0.002		
**2005–2010**	***t*/*t* + 1**	**HH**	**LH**	**LL**	**HL**	**Transition Type**	**Amount**	**Proportion**	**SF**	**SC**
	HH	2410	12	1	10	I	6413	0.972	0.027	0.973
	LH	24	307	25	0	II	86	0.013		
	LL	7	34	3472	32	III	94	0.014		
	HL	25	0	18	224	IV	8	0.001		
**2010–2015**	***t*/*t* + 1**	**HH**	**LH**	**LL**	**HL**	**Transition Type**	**Amount**	**Proportion**	**SF**	**SC**
	HH	2451	8	2	5	I	6490	0.983	0.016	0.984
	LH	13	330	9	0	II	46	0.007		
	LL	1	26	3475	14	III	61	0.009		
	HL	21	0	11	234	IV	3	0.000		
**2015–2018**	***t*/*t* + 1**	**HH**	**LH**	**LL**	**HL**	**Transition Type**	**Amount**	**Proportion**	**SF**	**SC**
	HH	2458	10	1	17	I	6487	0.983	0.017	0.983
	LH	9	332	23	0	II	46	0.007		
	LL	0	18	3461	18	III	66	0.010		
	HL	8	0	9	236	IV	1	0.000		

**Table 5:** Local Moran’s I transition matrix and spatiotemporal transition of ecosystem health at county scale.

**1990–1995**	***t*/*t* + 1**	**HH**	**LH**	**LL**	**HL**	**Transition Type**	**Amount**	**Proportion**	**SF**	**SC**
	HH	83	3	3	5	I	205	0.840	0.148	0.852
	LH	4	31	8	0	II	13	0.053		
	LL	0	8	78	4	III	23	0.094		
	HL	2	0	2	13	IV	3	0.012		
**1995–2000**	***t*/*t* + 1**	**HH**	**LH**	**LL**	**HL**	**Transition Type**	**Amount**	**Proportion**	**SF**	**SC**
	HH	77	4	4	4	I	190	0.779	0.201	0.799
	LH	5	26	11	0	II	19	0.078		
	LL	1	10	76	4	III	30	0.123		
	HL	5	0	6	11	IV	5	0.020		
**2000–2005**	***t*/*t* + 1**	**HH**	**LH**	**LL**	**HL**	**Transition Type**	**Amount**	**Proportion**	**SF**	**SC**
	HH	77	5	1	5	I	203	0.832	0.152	0.848
	LH	6	29	5	0	II	16	0.066		
	LL	3	6	85	3	III	21	0.086		
	HL	5	0	2	12	IV	4	0.016		
**2005–2010**	***t*/*t* + 1**	**HH**	**LH**	**LL**	**HL**	**Transition Type**	**Amount**	**Proportion**	**SF**	**SC**
	HH	84	3	1	3	I	205	0.840	0.148	0.852
	LH	8	26	6	0	II	14	0.057		
	LL	2	7	83	1	III	22	0.090		
	HL	6	0	2	12	IV	3	0.012		
**2010–2015**	***t*/*t* + 1**	**HH**	**LH**	**LL**	**HL**	**Transition Type**	**Amount**	**Proportion**	**SF**	**SC**
	HH	94	3	2	1	I	219	0.898	0.094	0.906
	LH	4	26	6	0	II	8	0.033		
	LL	0	4	88	0	III	15	0.061		
	HL	4	0	1	11	IV	2	0.008		
**2015–2018**	***t*/*t* + 1**	**HH**	**LH**	**LL**	**HL**	**Transition Type**	**Amount**	**Proportion**	**SF**	**SC**
	HH	95	3	0	4	I	222	0.910	0.086	0.914
	LH	3	26	4	0	II	8	0.033		
	LL	1	4	91	1	III	13	0.053		
	HL	1	0	1	10	IV	1	0.004		

## Data Availability

Not applicable.

## Supplementary Materials

The following supporting information can be downloaded at: https://www.mdpi.com/article/10.3390/ijerph192316144/s1, Table S1: Basic equivalent table of ecosystem service function value per unit area in the MRYR.
