# Supplementary material for: Multi-Scale Assessment and Spatio-Temporal Interaction Characteristics of Ecosystem Health in the Middle Reaches of the Yellow River of China
Source: Int J Environ Res Public Health. 2022 Dec 2;19(23):16144. doi: 10.3390/ijerph192316144 (PMC9739409; doi:10.3390/ijerph192316144)
Supplement: Supplementary file 1 [file ijerph-19-16144-s001.zip › ijerph-2033642-supplementary.pdf]

Supporting Information

List:

Table S1. Basic equivalent table of ecosystem service function value per unit area in the MRYR.

Table S1. Basic equivalent table of ecosystem service function value per unit area in the MRYR.

Service Function		Paddy Field	Mizuta	Forest	Lea	Wetlands	Water	Construction Land	Desert	Bare	Glacier
Provisioning service	Food production	1.36	0.85	0.31	0.38	0.51	0.80	0.00	0.01	0.00	0.00
	Raw material production	0.09	0.40	0.71	0.56	0.50	0.23	0.00	0.03	0.00	0.00
	Water supply	−2.63	0.02	0.37	0.31	2.59	8.29	0.00	0.02	0.00	2.16
Regulating service	Gas regulation	1.11	0.67	2.35	1.97	1.90	0.77	0.00	0.11	0.02	0.18
	Climate regulation	0.57	0.36	7.03	5.21	3.60	2.29	0.00	0.10	0.00	0.54
	Environmental cleaning	0.17	0.10	1.99	1.72	3.60	5.55	0.00	0.31	0.10	0.16
	Hydrological regulating	2.72	0.27	3.51	3.82	24.23	102.24	0.00	0.21	0.03	7.13
Supporting service	Soil conservation	0.01	1.03	2.86	2.40	2.31	0.93	0.00	0.13	0.02	0.00
	Maintain nutrient circulation	0.19	0.12	0.22	0.18	0.18	0.07	0.00	0.01	0.00	0.00
	Biodiversity	0.21	0.13	2.60	2.18	7.87	2.55	0.00	0.12	0.02	0.01
Cultural service	Provide aesthetic landscape	0.09	0.06	1.14	0.96	4.73	1.89	0.00	0.05	0.01	0.09
	All	3.89	4.01	23.09	19.69	52.02	125.61	0.00	1.1	0.2	10.27
